# Loneliness and the rate of motor decline in old age: the rush memory and aging project, a community-based cohort study

**DOI:** 10.1186/1471-2318-10-77

**Published:** 2010-10-22

**Authors:** Aron S Buchman, Patricia A Boyle, Robert S Wilson, Bryan D James, Sue E Leurgans, Steven E Arnold, David A Bennett

**Affiliations:** 1 Department of Neurological Sciences, Rush Alzheimer's Disease Center, Rush University Medical Center, 600 S. Paulina, Chicago, Illinois. 60612, USA; 2 Department of Behavioral Science, Rush Alzheimer's Disease Center, Rush University Medical Center, 600 S. Paulina Street, Chicago, Illinois. 60612, USA; 3 Departments of Psychiatry and Neurology, Center for Neurobiology and Behavior, University of Pennsylvania, 125 South 31st Street, Philadelphia, Pennsylvania 19104, USA

## Abstract

**Background:**

Being alone, as measured by less frequent social interactions, has been reported to be associated with a more rapid rate of motor decline in older persons. We tested the hypothesis that feeling alone is associated with the rate of motor decline in community-dwelling older persons.

**Methods:**

At baseline, loneliness was assessed with a 5-item scale in 985 persons without dementia participating in the Rush Memory and Aging Project, a longitudinal community-based cohort study. Annual detailed assessment of 9 measures of muscle strength and 9 motor performances were summarized in a composite measure of global motor function.

**Results:**

Linear mixed-effects models which controlled for age, sex and education, showed that the level of loneliness at baseline was associated with the rate of motor decline (Estimate, -0.016; S.E. 0.006, p = 0.005). For each 1-point higher level of loneliness at baseline, motor decline was 40% more rapid; this effect was similar to the rate of motor decline observed in an average participant 4 years older at baseline. Furthermore, this amount of motor decline per year was associated with about a 50% increased risk of death. When terms for both feeling alone (loneliness) and being alone were considered together in a single model, both were relatively independent predictors of motor decline. The association between loneliness and motor decline persisted even after controlling for depressive symptoms, cognition, physical and cognitive activities, chronic conditions, as well as baseline disability or a history of stroke or Parkinson's disease.

**Conclusions:**

Among community-dwelling older persons, both feeling alone and being alone are associated with more rapid motor decline, underscoring the importance of psychosocial factors and motor decline in old age.

## Background

Loss of motor function is a common consequence of aging and is associated with adverse health consequences[1-5]. The specific motor abilities impaired in old age vary and encompass a wide spectrum including loss of muscle strength and bulk, balance, dexterity and reduced gait speed which can occur even in the absence of overt diseases[6-8]. By 2030, 20% of Americans, roughly 72 million people, will be 65 years of age or older[9], and by the age of 80 years or older, the fastest growing segment, 40% or more will have some loss of motor abilities[10]. Identifying risk factors for age-related motor decline is an essential first step for the rational development of therapeutic interventions to reduce the growing burden of motor impairment in our rapidly aging population.

Although risk factors for common diseases known to cause motor dysfunction such as stroke are recognized, few risk factors for idiopathic motor decline in old age have been identified. While the benefits of physical activity on motor function is well-known[11-14], there is increasing recognition of the importance of lifestyle and psychosocial factors for healthy aging in older persons[15,16]. Increased social engagement as measured by the frequency of late-life social activities in older individuals is associated with longevity and a decreased risk of dementia, while being alone is associated with disability and a more rapid rate of motor decline[15-18].

Recent studies suggest that not only being alone, but also self-perceived isolation i.e., loneliness, has a detrimental effect on a wide range of physical functions including sleep, immune responses, level of physical activity, cognition and risk of Alzheimer's disease[19-23]. These reports suggest that not only being alone, but also loneliness might be related to motor decline in old age. Loneliness could serve as a marker for other processes such as inflammation or cardiovascular diseases which contribute to motor decline. Alternatively, loneliness may be a causal risk factor for motor decline. For example, since loneliness is associated with poor self-regulation, it may lead to behavioral changes such as decreased exercise or changes in eating habits which could in turn cause motor decline[24]. Furthermore, in addition to functional and structural links between social and motor behavior, social activity--like physical activity-may contribute to improved motor function by increasing neuronal plasticity and protecting against tissue damage[25]. Despite these reports, little is currently known about whether simply feeling lonely or disconnected from others and dissatisfied with social interactions is associated with motor decline in old age[23,24,26].

To test the hypothesis that feeling alone is associated with the rate of motor decline in old age, we used data from 985 older participants in the Rush Memory and Aging Project who underwent annual detailed examinations for up to 12 years[27]. At enrollment participants underwent assessment of loneliness with a modified version of the de Jong-Gierveld Loneliness Scale. They also underwent baseline and annual detailed exam which included assessment of motor strength and performances[18,23]. We used linear mixed-effect models to test the hypothesis that a higher level of loneliness at study entry was associated with a more rapid rate of motor decline during the course of the study. In further analyses, we examined whether including terms for both feeling alone and being alone (based on the frequency of participation in social activities and size of social network), showed separate effects with the rate of motor decline when considered together in a single model. Finally, we examined whether the association of loneliness and motor decline was confounded when controlling for depressive symptoms, cognition, other leisure activities and chronic conditions.

## Methods

### Participants

Participants were recruited from about 40 retirement facilities and subsidized housing facilities, as well as from church groups and social service agencies in northeastern Illinois. All participants signed an informed consent agreeing to annual clinical evaluation. In addition, all participants signed an anatomical gift act donating their entire brain and spinal cord, as well as selected nerves and muscles to Rush investigators at the time of death. The study was in accordance with the latest version of the Declaration of Helsinki and was approved by the Institutional Review Board of the Rush University Medical Center[27].

At the time of these analyses, 1201 participants had enrolled and completed a baseline evaluation. Eligibility for these analyses required 1) the absence of clinical dementia at the baseline evaluation; 2) a valid assessment of loneliness at baseline and 3) a baseline motor evaluation and at least one follow-up evaluation in order to assess change in motor function. We excluded 71 persons who met criteria for dementia at baseline and 86 persons who had completed a baseline evaluation but died before their first follow-up examination or had not been in the study long enough for follow-up evaluation. Of 1044 participants' eligible for these analyses 59 had missing data (5.7%). This left 985 persons for these analyses with a mean follow-up of 5.0 years (SD, 2.44; range 0.4, 12 years).

### Clinical Diagnoses

Clinical diagnoses were made using a multi-step process, as previously described[27]. Cognitive function testing included 19 performance tests which were summarized into a composite measure of global cognition [27]. Participants were then evaluated in person by an experienced physician who used published criteria to diagnose dementia[28], stroke[29], or Parkinson's disease [30].

### Assessment of Loneliness

We assessed loneliness using a modified version of the de Jong-Gierveld Loneliness Scale[23]. The 5 items included: a) "I experience a general sense of emptiness," b) "I miss having people around," c) "I feel like I don't have enough friends," d) "I often feel abandoned," and e) "I miss having a really good friend." Item scores were averaged to yield a total score that could range from 1 to 5, with higher values indicating a higher level of loneliness.

### Assessment of Motor Function

Grip and pinch strength were measured bilaterally using the Jamar hydraulic dynamometers (Lafayette Instruments, Lafayette, IN). Hand-held dynamometry (Lafayette Manual Muscle Test System, Model 01163, Lafayette, IN) was used to assess muscle strength in arm abduction, arm flexion, arm extension, hip flexion, knee extension, plantar flexion, and ankle dorsiflexion bilaterally. Time and number of steps to walk 8 feet and turn 360° were measured. Time to stand on each leg and then on toes for 10 seconds was recorded. We counted the number of steps off line when walking an 8 foot line in a heel to toe manner. We also measured the number of pegs that could be placed (Purdue pegboard) in 30 seconds and the rate of index finger tapping for 10 seconds (Western Psychological Services, Los Angeles, CA) bilaterally. A composite measure of global motor function was constructed by converting the raw score from each of the 18 motor measures to z scores using the mean and standard deviation from all participants at baseline and averaging z scores of all of the motor tests together [18].

### Assessment of Other Covariates

Two measures of social engagement were used as indicators of social isolation i.e. being alone. We used a previously established composite measure of late-life social activity in these analyses[23,31]. Frequency of participation in social activity was based on 6 items about activities involving social interaction. Each activity was rated on a 5-point scale with a higher number indicating higher frequency of participation with 1 indicating participation in the activity once a year or less; 2, several times a year; 3, several times a month; 4, several times a week; and 5, every day or almost every day. Responses on each item were averaged to yield the composite measure used in these analyses [23]. The second measure, social network size, quantified the number of children, family, and friends each person had and how often they interacted with them per month[32].

Sex was recorded at the baseline interview. Age in years was computed from self-reported date of birth, and date of the baseline clinical examination was that at which the strength measures were first collected. Education (reported highest grade or years of education) was obtained at the time of the baseline cognitive testing. Weight and height were measured and recorded at each visit by a trained technician blinded to previously collected data. Body mass index (BMI) was calculated as weight in kilograms divided by height in meters squared. Physical activity was assessed using questions adapted from the 1985 National Health Interview Survey[18]. Minutes spent engaged in each activity were summed and expressed as hours of activity/week. Frequency of participation in cognitively stimulating activities was quantified with a scale, wherein people rated how often they had participated in each of 7 cognitive activities (e.g., reading a newspaper) over the past year [33]. Disability was assessed at baseline with the 6-item Katz scale [34]. Depressive symptoms over the prior week were assessed with a 10-item version of the Center for Epidemiologic Studies Depression (CES-D) scale [35]. The sum of the number of vascular risk factors (i.e. the sum of hypertension, diabetes mellitus, and smoking), and vascular diseases (i.e., myocardial infarction, congestive heart failure, and claudication) were used in these analyses[36].

### Statistical Analyses

We examined the bivariate associations of loneliness and global motor with age, sex, education and other covariates. We used mixed-effect models [37] to assess the relation of loneliness with baseline level of global motor and its annual rate of change. The core model included terms for time in years since baseline as well as terms for loneliness at baseline which was centered at its mean and a term for its interaction with time since baseline. The term for time indicates the average annual rate of change in global motor scores for a typical participant with a median loneliness score; the term for loneliness indicates the average difference in motor function at baseline associated with a 1- point change in the level of loneliness score from the median; and the interaction of loneliness with time indicates the effect of a 1-point change in the level of loneliness score on the annual rate of change in global motor scores. To control for the effect of demographic variables, these and all subsequent models included terms for age, sex, and education and their interaction with time. In subsequent models, we added terms to determine if the association of loneliness and global motor scores might vary by age, sex, and education. Next we examined whether measures of social isolation or depression accounted for the association of loneliness with global motor scores. Then we examined whether several covariates which might affect motor function affected the association of loneliness and motor decline. To determine the clinical significance of the amount of change in global motor function, we constructed Cox proportional hazards models examining adverse health consequences of change in motor function and estimated the hazard ratios associated with a given unit of change. These models controlled for age, sex, education, and baseline global motor function. For these analyses we used ordinary least squares regression to estimate the annual rate of change in global motor function for each person. Models were examined graphically and analytically and assumptions were judged to be adequately met. *A priori *level of statistical significance was 0.05. Programming was done in SAS version 9.1.3 (SAS Institute Inc, Cary, NC) [38].

## Results

### Descriptives of Loneliness

The characteristics of the cohort at baseline are included in Table 1. Baseline loneliness scores were approximately normally distributed (mean, 2.26; SD, 0.65; Q_1-3_, 0.60). Scores ranged from 1.0 to 4.6 with higher values indicating more loneliness. Loneliness did not vary by sex (t [983] = -1.29, p = 0.199). Participants, who reported higher levels of loneliness at baseline were older, less educated, reported less frequent participation in social, physical, and cognitive activities, reported more disability, had lower cognitive function, and were more likely to have vascular diseases (Table 2).

**Table 1:** Demographics of the Cohort at Baseline (N = 985)*

Variable	Mean (SD)
Age (years)	79.67 (7.36)
Sex (% male)	N = 245 (24.87%)
Education (years)	14.42 (3.14)
Social Activity	2.61 (0.58)
Social Network Size	6.59(5.83)
Physical Activity (hrs./week)	3.17( 3.71)
Cognitive Activity Score	3.15 (0.69)
Katz Disability	0.20 (0.67)
Global Cognition	0.11 (0.54)
Depressive Symptoms Score	1.33 (1.78)
BMI (kg/m^2^)	27.35 (5.25)
Vascular Risk Factors (sum)	1.15 (0.80)
Smoking	N = 395 (40.14%)
Diabetes	N = 132 13.40%
Hypertension	N = 603 (61.22%)
Vascular Diseases (sum)	0.35 (0.63)
Myocardial Infarction	N = 112 (11.38%)
Congestive Heart Failure	N = 41 (4.73%)
Claudication	N = 75 (7.61%)
Stroke	N = 82 (8.3%)
Parkinson's Disease	N = 13 (1.3%)

*MMSE: Mini-Mental State Examination (range: 18-30), a higher score indicates a higher level of cognition. Social Activity: Self-reported frequency of participation in six social activities a higher score indicates more frequent participation. **Social Activity**: Self-reported frequency of participation in 6 items about activities involving social interaction, a higher score indicates more frequent participation; **Social Network Size**: Self-reported number of children, family, and friends each person had and how often they interacted with them per month **Physical Activity**: Self-reported frequency of participation in 5 physical activities (hours/week), a higher score indicates more frequent participation. **Cognitive Activity**: Self reported frequency of participation in 7 cognitive activities, a higher score indicates more frequent participation. **Katz Disability**: 6 item measures of basic activities of daily living, a higher score indicates greater disability. **Global Cognition**: Composite measure of cognition based on performances on 18 cognitive tests, a higher score indicates a higher level of cognition. **Depressive Symptoms**: Modified 10 item CESD scale, a higher score indicates greater depressive symptomatology. **BMI**: Body mass index: weight in kilograms divided by height in meters squared. **Vascular Risk Factors**: sum of smoking, diabetes, and hypertension self-reported. **Vascular Diseases**: sum of myocardial infarction, congestive heart failure, claudication and stroke self-reported.

**Table 2:** Correlations of Baseline Global Motor Score and Loneliness with Other Covariates

Variable	Global Motor Score	Loneliness
**Age**	-0.44**	0.17**
**Education**	0.17**	-0.21**
**Social Activity**	0.27**	0.20**
**Social Network**	0.11**	-0.24**
**Global Cognition**	0.32**	-0.26**
**Depressive Symptoms**	-0.23**	0.37**
**Physical Activity**	0.22**	-0.04
**Cognitive Activity**	0.26**	-0.22**
**Katz Disability**	0.36**	0.11**
**Body Mass Index**	-0.02	0.05
**Vascular Diseases**	-0.18**	0.03
**Vascular Risk Factors**	-0.02	0.01

** = p < 0.001

### Loneliness and Change in Motor Function

Baseline global motor scores ranged from -2.11 to 2.09 (mean -0.06; SD, 0.59; Q_1-3 _0.85). Men had higher global motor scores (mean, 0.19; SD, 0.60) than women (mean, -0.14; SD, 0.56) [t [983] = -7.95, p < 0.001]. Participants with higher global motor scores were younger and better educated, reported a larger social network; with more frequent social, cognitive and physical activities, less disability, better cognition, less depressive symptoms and vascular diseases (Table 2).

**Figure 1:**
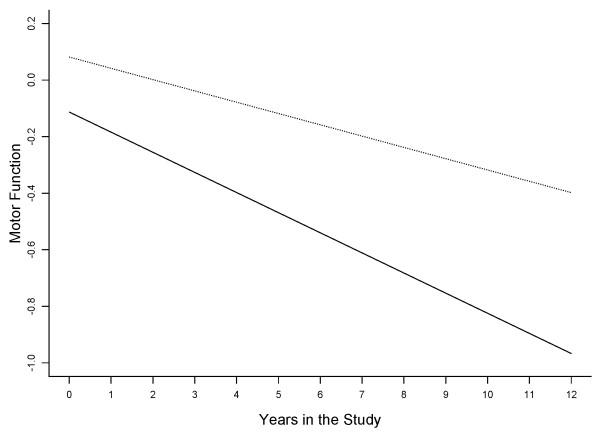
**Loneliness and the Rate of Motor Decline**. This model derived (Model A, Table 3) figure illustrates the rate of motor decline in two participants one who is lonely (solid line: 90^th ^percentile, score = 3.2) and the second not lonely (dotted line: 10^th ^percentile, score = 1.4).

We used a linear mixed effect model controlled for age, sex, and education to test the hypothesis that baseline loneliness score is associated with the rate of motor decline. On average, global motor declined by about -0.04 unit/year (Time, Table 3, Model A). Baseline loneliness was associated with the global motor score at baseline (Loneliness, Table 3) as well as the annual rate of change in global motor score (Loneliness*Time, Table 3, Model A). A 1- Comparing the rate of motor decline in two participants with different loneliness scores at baseline, shows that the person with a 1-point higher loneliness score would exhibit a 40% more rapid annual rate of motor decline. This can be computed by dividing the estimate for the interaction term of loneliness and rate of motor decline (Loneliness * Time, Table 3, Model A) by the estimate for the term for the annual rate of motor decline (Time, Table 3, Model A). Figure 1, based on this model, compares the rate of motor decline in two participants with high and low baseline loneliness scores. The rate of motor decline for the lonely person (90^th ^percentile, score, 3.2) declined about 80% more rapidly as compared to a person who was not lonely (10^th ^percentile, score, 1.4).

**Table 3:** Loneliness Is Associated with the Rate of Change in Motor Function

Terms	Model A*	Model B**
Time	-0.039 (0.013 p =,0.004)	-0.088 (0.024, p < 0.000)
Age	-0.037 (0.002, p < 0.001)	-0.034 (0.002, p < 0.001)
Age × Time	-0.004 (0.001, p < 0.001)	-0.004 (0.001, p < 0.001)
Sex	0.394 (0.038, p < 0.001)	0.425 (0.037, p < 0.001)
Sex × Time	-0.077 (0.009, p < 0.001)	-0.076 (0.009, p < 0.001)
Education	0.021 (0.005, p < 0.001)	0.015 (0.005, p = 0.004)
Education × Time	0.0004 (0.001, p = 0.722)	-0.0001 (0.001, p = 0.955)
Loneliness	-0.103 (0.026, p < 0.001)	-0.076 (0.026, p = 0.003)
Loneliness × Time	-0.016 (0.006, p = 0.005)	-0.014 (0.006, p = 0.018)
Social Activity		0.224 (0.029, p < 0.001)
Social Activity × Time		0.016 (0.007, p = 0.022)
Social Network		-0.001 (0.003, p = 0.725)
Social Network × Time		0.0004 (0.0001, p = 0.519)

* Model A is based on a linear mixed-effect model which shows the cross sectional association of a 1-point score on the Loneliness scale with the baseline global motor score (Loneliness) as well as its association with the rate of change in motor function (Loneliness × Time) and controls for age, sex and education and their interaction with Time. Units of comparison: Time in years since baseline, Age and education in years. ** Model B includes all the same terms as Model A but also includes terms to control for two measures of social isolation ( frequency of late-life social activities and social network size and their interactions with Time).

Since the term for age in the core model was also related to the rate of global motor decline, we could compare the amount of motor decline associated with increased age with the amount of motor decline associated with loneliness. For each additional year of age, global motor score declined an additional 0.004 standard units (Age*Time, Table 3, Model A). In contrast, each 1 point increase in baseline loneliness was associated with an additional 0.016 standard unit decline in global motor (Loneliness*Time, Table 3, Model A). Thus, a 1-point higher loneliness score was equivalent to an average participant being about 4 years older at baseline. Additional analyses showed that the association of loneliness with motor decline (Loneliness *Time) did not vary by age, sex or education (results not shown).

### Loneliness, Social Isolation and Change in Motor Function

Indicators of social isolation such as frequency of social activity have been associated with disability, mortality and motor decline as previously reported[18]. Therefore, we repeated the core model adding terms for social isolation (i.e., late-life social activity and social network size) as well as their interaction with the annual rate of motor decline (Time). In this analysis, both loneliness and social isolation as measured by the frequency of social activities were relatively independently associated with the rate of motor decline (Table 3, Model B). Social network size was not related to motor function or its rate of decline in this same model (Table 3, Model B).

### Loneliness, Other Covariates and Change in Motor Function

Because feeling lonely can be a symptom of depression and lonely persons are prone to experience depressive symptoms, we conducted additional analyses in an effort to disentangle these related constructs. In these analyses, we excluded 1 item about loneliness (ie, "I felt lonely") from the 10-item CES-D scale (9-item CES-D scale, mean, 1.15 SD, 1.57). Controlling for the 9-item CES-D score in the core model did not reduce the association of loneliness with motor decline [Loneliness *Time, Estimate, -0.018 (S.E. 0.006, p = 0.002)]. Including a term for global cognition in the core model reduced the association of loneliness with motor decline by about 18%, but the association remained significant (Loneliness * Time, Estimate, -0.013 (S.E. 0.005, p = 0.015). In subsequent analyses including terms for the frequency of cognitive and physical activities, body composition, vascular risk factors and vascular disease burden in combination with the other terms included in model A (Table 3) described above did not affect the association of loneliness and the rate motor decline (results not shown).

Next we determined that our results were not due to participants with baseline disability or a history of motor disorders due to neurologic disorders. The association between loneliness and the rate of motor decline was unchanged when we controlled for baseline disability using the Katz scale (Loneliness *Time, Estimate, -0.017 (S.E. 0.005, p = 0.001) or after excluding participants with a history of stroke or Parkinson's disease (Loneliness *Time, Estimate, -0.015 (S.E. 0.006, p = 0.005).

### Clinical Significance of the Change in Motor Function Associated with Loneliness

To determine the clinical significance of the increased rate of decline of global motor scores associated with a 1-point increased loneliness score at baseline (Loneliness * Time, Model A, Table 3), we constructed Cox proportional hazards models examining the association of change in motor function with death and subsequently estimated the hazard ratios associated with a 40% increased annual decline, (i.e., the amount of change in global motor scores associated with a 1-point higher baseline loneliness score). From these models (data not shown), we calculated that the 40% increased rate of motor decline in a participant with a 1-point higher loneliness score at baseline was associated with about a 50%% increased risk of death as compared to a participant with an average loneliness score (Hazard Ratio: 1.21; 95% CI: 1.08, 1.35).

## Discussion

In a cohort of nearly 1000 older persons free of dementia at baseline, we found that a higher level of loneliness (i.e., self-perceived isolation) was associated with a more rapid rate of motor decline in community-dwelling elders. This association persisted even after controlling for social isolation as measured by frequency of social activities and social network size, as well as a wide range of potential confounding variables including depression, cognition, physical and cognitive activities and chronic conditions. In several sensitivity analyses, this association was unchanged after controlling for baseline disability as well as a history of stroke or Parkinson's disease.

Accumulating evidence suggests that social isolation as measured by frequency of late-life social activities or size of social network is related to adverse health outcomes such as longevity and risk of dementia, as well as the rates of cognitive and motor decline[18,39]. However, not only social isolation but also self-perceived isolation i.e., loneliness, has a detrimental effect on a wide range of functions including sleep, immune responses, level of physical activity, cognition and risk of AD[19-23]. A prior study reported that loneliness is associated with decreased physical activity or exercise, but this report analyzed physical activity levels which were based on self-report and did not assess levels of other late-life leisure activities[24]. The current study extends prior reports in several important ways. First we report that loneliness is related to the rate of motor decline derived from objective motor performances tested annually for up to 12 years. Second, we show that when self-perceived isolation and social engagement as measured by late-life social activities are considered together in the same model, both are relatively independent predictors of the rate of change in motor function. Third, the association between loneliness and motor decline persisted even after controlling for a wide range of leisure activities including social, physical and cognitive activities, depressive symptoms and other possible confounding covariates as well as after controlling for baseline disability or history of stroke and PD. These results have important translational implications because they suggest that public health interventions designed to maintain motor function in older adults need to consider the possible role of self-perceived isolation as a modifiable risk factor, which might increase the efficacy of other efforts implemented to decrease the burden of age-related motor decline.

The basis for the association between loneliness and motor decline is uncertain. Human social behavior is generated in the brain through interconnected brain structures which process different elements of sociocognitive and socioaffective information which are eventually integrated and translated into motor action[40]. Loneliness and motor decline may be associated since both depend on the structural and functional integrity of neural systems underlying the initiation, planning and execution of motor action and might both be affected by common pathophysiological processes. Moreover, recent work suggests that mirror neurons are thought to play important roles not only for generating movement but also for a wide range of activities essential for social interaction including self-awareness and empathy. Further work is needed to elucidate the role of mirror neurons in human behavior, but this raises an intriguing possibility that mirror neurons might provide a structural causal linkage between self-perceived isolation i.e., loneliness and motor actions[41]. Motor function is necessary for social behavior and is thus an integral component of one's social body. Recent work suggests that social pain may function as an aversive signal, like physical pain, signaling the need to take action against factors which can damage or harm one's social body[42]. Thus, loneliness, as an expression of social pain, may be associated with motor decline because it serves as an aversive signal for factors which may impair motor function and the capacity for social behavior.

Loneliness may represent a true risk factor which causes motor decline. For example, loneliness is associated with poor self-regulation which may lead to behavioral changes such as decreased exercise or changes in eating habits causing motor decline[24]. Alternatively, there may be common pathophysiological processes which affect both loneliness and motor impairment in old age. Loneliness is associated with a wide range of physiologic changes such as higher levels of cortisol, increased inflammation, immune dysfunction, increased cardiovascular disease and impaired sleep patterns which may all contribute to both loneliness and motor decline [20,21,43-45]. In addition, to the functional and structural links between social and motor behavior, it is noteworthy that the benefits of social activity--like physical activity-may contribute to improved motor function by increasing neuronal plasticity and protecting against ischemic or neurotoxic damage[25]. Animals subjected to social isolation show decreased dendritic arborization in the hippocampus and prefrontal cortex and down-regulation of brain-derived neurotrophic factor which may be associated with impaired plasticity degrading the ability to compensate for the accumulation of age-related pathologies[42]. Similar findings can be seen in humans with decreased levels of physical activity which also is related to the motor decline in old age. Finally, recent work suggests that loneliness is associated with alteration in human genome-wide transcriptional activity that might account for increased inflammatory diseases in loneliness[44,45]. The current cohort study cannot distinguish between the existence of a pathophysiological process affecting both loneliness and motor decline or the possibility that motor decline that is caused by loneliness. Thus, further work is needed to clarify the neurobiology underlying the association between loneliness and age-related motor decline as well as the degree to which other psychosocial factors may contribute to motor decline in the elderly.

Our study has some limitations. Most importantly, inferences regarding causality must be drawn with great caution from observational studies. While the findings were robust to potential confounding variables and sensitivity analyses, the potential for reverse causality cannot be excluded. Further, it is possible that residual confounding from an unmeasured latent variable is related to both loneliness and motor decline. Other limitations include the selected nature of the cohort, the self-report measures of chronic diseases and leisure activities and that this study did not assess simultaneous change in both loneliness and motor decline.

However, several factors increase confidence in our findings. Perhaps most importantly, the study enjoys high follow-up participation reducing bias due to attrition. In addition, loneliness was assessed among persons without dementia based on a detailed clinical evaluation and motor function was evaluated as part of a uniform clinical evaluation and incorporated many widely accepted and reliable strength and motor performance measures; strength testing was done in all four extremities, and motor performances were tested in both the arms and legs. The aggregation of multiple measures of motor function into a composite measure yields a more stable measure of motor function and increases statistical power to identify associations. In addition, a relatively large number of older persons representative of the general population were studied, so that there was adequate statistical power to identify the associations of interest while controlling for several potentially confounding variables.

## Conclusions

In a cohort of nearly 1000 community-dwelling older persons free of dementia at study entry and followed for up to 12 years, we found that simply feeling lonely or dissatisfied with social interactions is associated with a more rapid rate of motor decline. Furthermore, we found that both feeling alone and being alone are associated with a more rapid rate of motor decline. These findings underscore that psychosocial factors may not only affect the efficacy of interventions designed to maintain motor function in older adults but that these factors such as self-perceived isolation might also be modifiable risk factors that can be targeted to increase the efficacy of efforts to meet the growing public health challenge and burden of motor impairment in our rapidly aging population.

## Competing interests

The authors (A. S. Buchman, MD, P. A. Boyle, PhD, R.S. Wilson, PhD, B. James, PhD, S. E. Leurgans, PhD, Arnold, MD and D.A. Bennett, MD) have no conflicts of interest to report.

## Authors' contributions

ASB, MD had full access to all the data in the study and takes responsibility for the integrity of the data and the accuracy of the data analysis and affirms that everyone who contributed significantly to the work has been listed. He was involved with study concept and design, analysis and interpretation of data, and preparation of the manuscript. Drs. ASB, PAB, RSW, BDJ, SEL, SEA, and DAB were involved in study concept, acquisition of data, assisted with the analysis and interpretation of the data, and critically revised the manuscript for important intellectual content. All authors have seen and approved the final version.

## Pre-publication history

The pre-publication history for this paper can be accessed here:

http://www.biomedcentral.com/1471-2318/10/77/prepub
